# Molecular Cloning and Characterization of SYCP3 and TSEG2 Genes in the Testicles of Sexually Mature and Immature Yak

**DOI:** 10.3390/genes10110867

**Published:** 2019-10-30

**Authors:** Qudratullah Kalwar, Min Chu, Anum Ali Ahmad, Xiaoming Ma, Renzheng Zhang, Fulong Ma, Jianpeng Xie, Xuezhi Ding, Xiaoyun Wu, Pengjia Bao, Ping Yan

**Affiliations:** 1 Key Laboratory of Yak Breeding Engineering, Lanzhou Institute of Husbandry and Pharmaceutical Sciences, Chinese Academy of Agricultural Science, Lanzhou 730050, Gansu, China; qudratullahkalwar@gmail.com (Q.K.); chumin@caas.cn (M.C.); anum2017@lzu.edu.cn (A.A.A.); zhangrenzheng51@163.com (R.Z.); mafulong2018@outlook.com (F.M.); 18709480641@163.com (J.X.); dingxuezhi@caas.cn (X.D.); wuxiaoyun@caas.cn (X.W.); baopengjia@caas.cn (P.B.); 2 Department of Animal Reproduction Shaheed Benazir Bhutto, University of Veterinary and Animal Sciences, Sakrand 67210, Sindh, Pakistan; 3 State Key Laboratory of Grassland Agro-Ecosystems, School of Life Sciences, Lanzhou University, Lanzhou 730050, Gansu, China

**Keywords:** Yak, spermatogenesis, cloning, gene expression, SYCP3, TSEG2

## Abstract

Testis-specific genes play an essential part in the centromere union during meiosis in male germ cells, spermatogenesis, and in fertility. Previously, there was no research report available on the expression pattern of *SYCP3* and *TSEG2* genes in different ages of yaks. Therefore, the current research compared the expression profiling of *SYCP3* and *TSEG2* genes in testes of yaks. The expression pattern of *SYCP3* and *TSEG2* mRNA was investigated using qPCR, semi-quantitative PCR, western blot, immunohistochemistry, and molecular bioinformatics. Our findings displayed that *SYCP3* and *TSEG2* genes were prominently expressed in the testicles of yaks as compared to other organs. On the other hand, the protein encoded by yak *SYCP3* contains Cor1/Xlr/Xmr conserved regions, while the protein encoded by yak TSEG2 contains synaptonemal complex central element protein 3. Additionally, multiple alignments sequences indicated that proteins encoded by Datong yak SYCP3 and TSEG2 were highly conserved among mammals. Moreover, western blot analysis specified that the molecular mass of SYCP3 protein was 34-kDa and TSEG2 protein 90-kDa in the yak. Furthermore, the results of immunohistochemistry also revealed the prominent expression of these proteins in the testis of mature yaks, which indicated that SYCP3 and TSEG2 might be essential for spermatogenesis, induction of central element assembly, and homologous recombination.

## 1. Introduction

The synaptonemal complex (SC) is an evolutionarily conserved protein assembly that holds together homologous chromosomes during prophase of the first meiotic division [1]. In addition, SC is vital for the formation of crossing over phenomenon and meiotic recombination [2]. Several studies reported that meiotic failure, infertility, and embryonic death in mice resulted due to disruption of SC formation [3,4,5]. In humans, the unreliable formation of SC has been associated with infertility, miscarriage, and Down’s syndrome [6]. The lateral elements *Sycp2* and *Sycp3* and central element Syce1, Syce2, Syce3 (TSEG2), and Tex12 protein genes are the main constituents of synaptonemal complex [7,8,9]. If any error happens during meiosis, it can lead to a disorder that may cause an irregular number of chromosomes [10]. Before the first meiotic division, the main stage in meiosis is the pairing of two homologous chromosomes [11]. When synapsis is finished, the homologous chromosomes go through normal recombination [12]. The synaptonemal complex brings the homologous chromosomes in close association and causes synapsis regulation. However, the major component of the synaptonemal complex is *SYCP3* and *TSEG2* which plays a crucial part in meiosis of spermatogenesis, fertility and homologous chromosome pairing in males [13]. Syce3 is essential for fertility in males and females; meiotic arrest occurs due to damage of Syce3 and results in the beginning of synapsis obstruction. Schramm et al. [9] evaluated that Syce3 is essential for induction of central element association and homologous recombination. The crucial step in meiosis is the formation of the synaptonemal complex, which assists in synapsis and homologous chromosome orientation. Infertility may occur when the incorrect union of homologous chromosomes happens. Jing et al. [14] found that functional Syce3 most likely forms a dimer or higher-order oligomer in cells. The Syce3 N-helix interrelates with the Syce1 C-helix, which is another central element component, and packing of helical may facilitate the association of each central element protein component, and plays an essential role in forming synaptonemal complex central elements. The *TSEG-2* gene is also well-known as synaptonemal complex central element 3(Syce3) and present in spermatogonia and spermatocytes. The testis-specific genes are essential for meiosis and for better treatment of male infertility as reported in mouse testis [15]. The expression of *TSEG2* was high in the testis of mice; however, different expression during postnatal development stages of testis correlated with mouse sexual maturation and spermatogenesis [16]. Therefore, *TSEG2* gene may play a vital role in the apoptosis of spermatogenic cells and pathogenesis of cryptorchidism [15]. The previous study identified that TSEG2 proteins are essential for synapsis and synaptonemal complex formation in mice [17]. Moreover, Yuan et al. [18] revealed that the failure of SC formation and synapsis were due to disorder of *SYCP3,* which results in a sexually dimorphic phenotype and complete infertility in mice. Numerous *SYCP3* alterations have been observed in infertile women and men at the clinical level [19]. Furthermore, Wang et al. [20] reported significantly high mRNA expression level of bovine *SYCP3* in testes. This suggests that bovine *SYCP3* gene plays prominent part in meiosis of spermatogenesis and the expression is influenced by inter specific hybridization among cattle and yak [20]. Additionally, Shi et al. [21] perceived that during meiosis *SYCP3* gene was involved in sex, body maintenance, DNA recombination, and synaptonemal complex formation.

Until now, most reports on *SYCP3* and *TSEG2* gene in male mammals have mainly focused on mouse, human and cattle, but the regulatory mechanisms are not precisely the same in different species. The yak is an economically-important livestock animal in agriculture; however, little is known about the expression profiles and biological functions of *SYCP3* and *TSEG2* in yak testes. Hence, it is essential to explore the mechanisms of yak spermatogenesis by investigating the expression patterns and regulatory roles of *SYCP3* and *TSEG2* in different developing stages of yak testes. 

## 2. Materials and Methods 

This research was conducted between 2018 and 2019 at the Key Laboratory of Yak Breeding Engineering, Lanzhou Institute of Husbandry and Pharmaceutical Sciences, Chinese academy of agricultural Sciences, Lanzhou 730050, Gansu, China. All the samples were collected from the Gansu breeding cooperatives and from Qinghai province, China. Sample collection was performed in strict accordance with the guide for the Care and Use of Laboratory Animals, Lanzhou Institute of Husbandry and Pharmaceutical Sciences, China. Additionally, all the animals were slaughtered under anesthesia, and all necessary efforts were made to minimize the risk of suffering. The legal certificate number is SCXK (Gan) 2014-0002. Furthermore, the animals were separated into four groups (6 months, 18 months, 30 months, and 6 years). Every age group contained four male yaks. The heart, testis, kidney, liver, lung, intramuscular fat, and spleen samples were collected from animals. The excised tissues were frozen in liquid nitrogen for transport and ultimately stored at −80 °C.

### 2.1. Total RNA Extraction and cDNA Synthesis 

The Total RNA extraction was done by using Trizol method (Tri Pure Isolation Reagent, Roche, Indianapolis, IN, USA), following the instructions of the manufacturer. The cDNA synthesis from extracted RNA was done by using Prime Script kit (Takara, Dalian, China). For cloning of the *SYCP3* and *TSEG2,* the cDNA was reverse transcribed for 30 min at 42 °C, followed by incubation for 5 s at 85 °C. While the cDNA which was used for the analysis of semi-quantitative PCR was reverse transcribed at 37 °C for 15 min and final incubation at 85 °C for 5 s.

### 2.2. Molecular Cloning

The whole open reading frame sequence of *SYCP3*, *TSEG2* and *GAPDH* of bovine was gotten from http://www.ncbi.nlm.nih.gov/genbank/. Premier 5.0 software was used for designing the primer for PCR and qPCR and primers were synthesized from Takara (Dalian, China). The cloning was carried out with 50 µL polymerase chain reaction; it comprised 25 µL one-shot LA PCR Mix, 1 µL template, 1 µL each forward and reverse primer and sterilized water 28 µL. The polymerase chain reaction was done using the following cycling conditions: 95 °C for 1 min; followed by 30 cycles of 95 °C for 10 s, annealing at 68 °C for 15 min, and 72 °C for 90 s and extension at 72 °C for 15 min. The reaction products were separated on 1% agarose gel. Lastly, for the purification of target bands, gel extraction kit (TIANGEN, Beijing, China) was used, and purified products were cloned into the pMD19-T vector and sequenced by GENEWIZ, Beijing, China. 

### 2.3. Exploration through Semi-Quantitative PCR

The semi-quantitative PCR reaction for characterization of *SYCP3* and *TSEG2* in different tissues comprised a total of 162.5 µL Taq PCR master mixture, 13 µL each primer (forward and reverse), and ddH_2_O 123.5 µL after that reaction mixture were divided into 24 µL aliquots into 11 tubes. Additionally, cDNA obtained from ten kinds of tissues was used, 1 µL of the cDNA was added into the ten tubes, and one tube as a control. The reaction was conducted according to Yan et al. [22]. The PCR was implemented using the different conditions: for 3 min at 94 °C; followed by 30 cycles of 94 °C for 30 s, annealing at 60 °C (for GAPDH, SYCP3 and TSEG2) for 30 s, and 72 °C for 1 min; and extension at 72 °C for 5 min. The reaction products were visualized on 1 % agarose gel stained with ethidium bromide.

### 2.4. Exploration through Quantitative Real-Time PCR

The PCR reaction for quantitative real-time PCR contained 12.5 µL TB Green premix Ex Taq II (Tli RNaseH Plus) (TAKARA, Beijing, China), 1 µL of 10 mM each forward and reverse primers, 8.5 µL ddH2O, and cDNA template 2 µL. The polymerase chain reaction was executed with a Thermal cycler Dice Real Time System (Bio-Rad, Hercules, CA, USA) with different cycling conditions as 95 °C for 30 s followed by 39 cycles of 95 °C for 5 s and annealing for 30 s at 60 °C.

### 2.5. Primer Designing and Bioinformatics Analysis 

Primers were designed for cloning and expression of gene using the Primer-BLAST web tool (https://www.ncbi.nlm.nih.gov/tools/primer-blast/index.cgi?LINK_LOC=blastHome). The open reading frame finder program (http://www.ncbi.nlm.nih.gov/gorf/gorf.html) was implemented to predict the amino acid sequence of SYCP3 and TSEG2. While the conserved domain of SYCP3 TSEG2 protein was anticipated by using Pfam (http://pfam.janelia.org/). The three dimensional and secondary structures for the protein encoded by SYCP3 and TSEG2 were examined by using the PDB viewer, PSI pred (http://bioinf.cs.ucl.ac.uk/psipred) and SWISS-MODEL (http://swissmodel.expasy.org/). (see Table 1).

### 2.6. Exploration through Tunnel Assay

For tunnel assay, slices were incubated for l0 min with l0 µg/mL proteinase K in PBS at ambient temperature, additionally; streptavidin HRP solution was diluted to 1:1000 in PBS, after that, the nuclear staining in apoptotic cells were recognized by using the tunnel apoptosis assay kit (Boster, Wuhan, China) according to the manufacturer’s guideline. The apoptotic index was calculated as the percentage of cells that tested TUNEL-positive [23].

### 2.7. Tissue Immunostaining

A sodium citrate solution was used to retrieve antigenicity of deparaffinized sections. Then slides were blocked into five percent serum in PBS for one hour at 25 °C and incubated at 4 °C for whole night in Anti SCP3 antibody (diluted in 1:500; Abcam, Cambridge, UK). Consequently, HRP Conjugated Anti Rabbit Secondary antibody was used for staining of sections and diluted in 1:10,000 (Jackson, MS, USA), finally microscope Leica, GER was used for capturing of the pictures.

### 2.8. Western Blotting

The proteins were extracted from the testis similarly as defined earlier [22]. The protein concentration was estimated by using a protein assay kit (Beyotime, Shanghai, China). Firstly, proteins were fixed in 12% Tricine SDS PAGE for gel electrophoresis and then transferred onto PVDF membranes (Roche, Indianapolis, IN, USA), after blocking in phosphate buffered saline tween 20 containing 5% nonfat milk, then membranes were incubated at 4 °C for whole night with anti SCP3 and anti SYCE3 antibody (1:1000, Abcam, Cambridge, UK) and anti β-actin (1:1000; Abcam, Cambridge, UK). After washing the membranes were incubated with goat anti rabbit IgG/HRP antibody (1:5000, Bioss, Beijing, China) for 1 hour, and finally bands intensity was determined by using ECL detection system (Pierce, Appleton, WI, USA).

### 2.9. Statistical Analyses 

Each trial was repeated in at least 3 replicates and the quantitative expression level of each target gene was calculated by using threshold cycle 2^-ΔΔCt^ method [24]. The significance of the differences in data was assessed using ANOVA. Next, the immune-histomorphometry of the stained tissue sections was measured by Image Pro v10 (International Scientific Community, Anaheim, CA, USA) and analyzed statistically with Graph-Pad Prism 7.0software (IBMP Crop, Armonk, NY, USA). The results are presented as the mean ± SEM. The statistical significance of differences between the means was also analyzed by t-test (*p* < 0.05). 

## 3. Results

### 3.1. Expression Profile of SYCP3 and TSEG2 by Semi-Quantitative PCR and Quantitative Real Time PCR 

**Figure 1:**
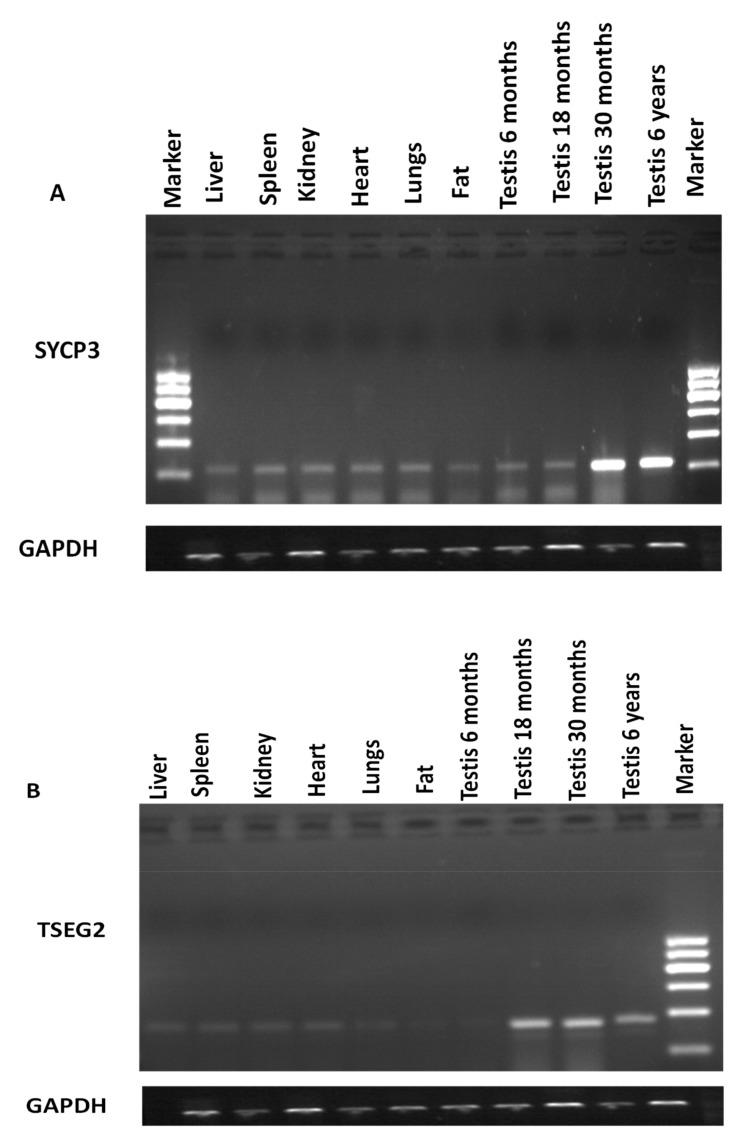
Analysis of the *SYCP3* and *TSEG2* mRNA using semi-quantitative PCR from different tissues of Datong yak (**A**) *SYCP3* expression (**B**) *TSEG2* expression.

**Figure 2:**
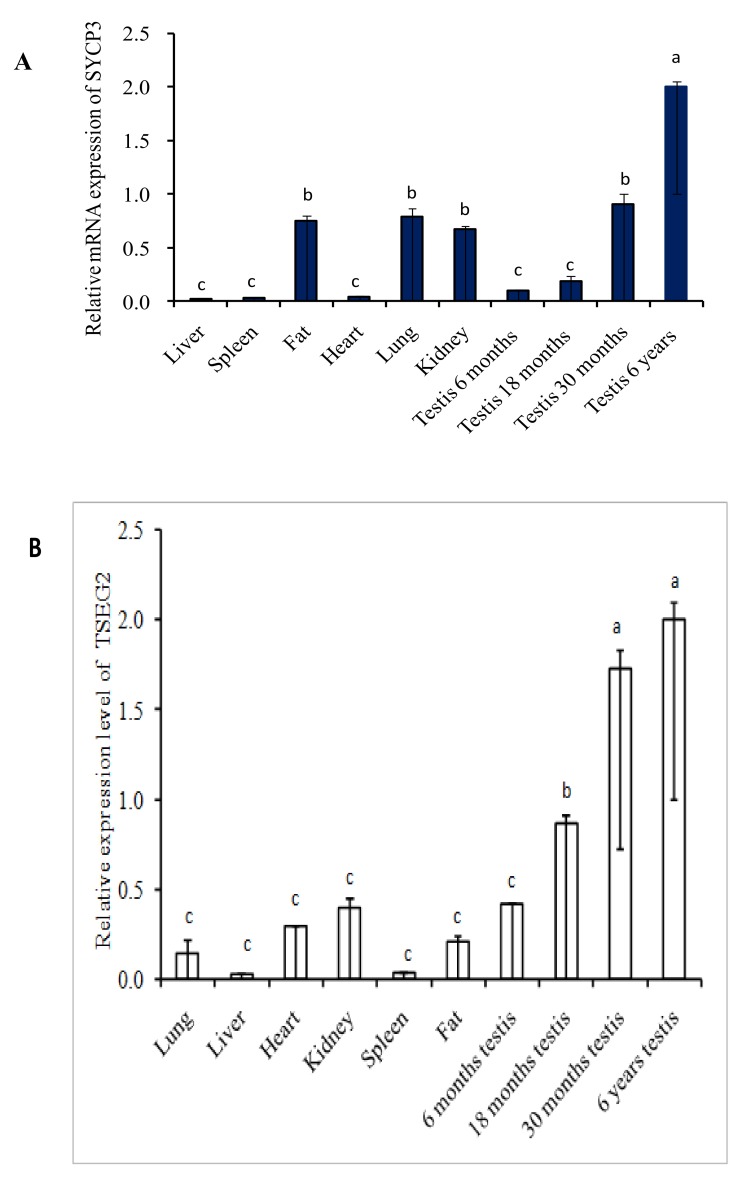
The quantitative real time PCR was used for assessment of the expression of SYCP3 and TSEG2 mRNA (**A**) SYCP3 (**B**) TSEG2.

The expression level of *SYCP3* and *TSEG2* from different tissues of yak was evaluated by semi-quantitative PCR. The outcomes of the semi-quantitative PCR indicated that the expression level of *SYCP3* and *TSEG2* mRNA was higher in the testis of yak as compared to other organs (Figure 1). As we compared testis of different ages, expression was low in 6 months and 18 months as compared to 30 months and 6 years testis. We also analyzed the expression pattern of *SYCP3* and *TSEG2* mRNA by performing quantitative real-time PCR in different tissues of Yak (Figure 2). These results denoted that the expression level *TSEG2* mRNA was low in spleen and liver, moderate in lungs, heart, kidney, and sub cutaneous fat, but was significantly higher in the testicles.

### 3.2. Structures of SYCP3 and TSEG2 Gene

**Figure 3:**
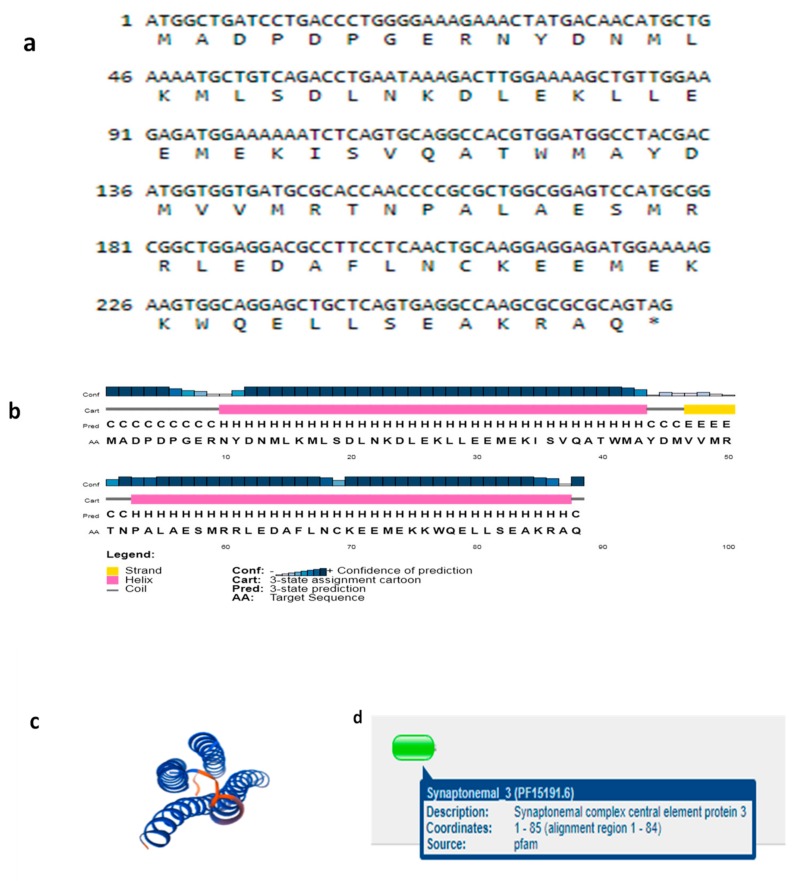
Exploration of the protein sequence encoded by TSEG2 from Datong yak. The protein encoded by Datong Yak TSEG2 contains a synaptonemal complex central element 3. (**a**) The sequences of yak *TSEG2* genes and predicted protein. (**b**) Estimated secondary structure for the protein encoded by TSEG2 has a long vertical bar, short vertical bar, coil a-helix; sub medium vertical bar b turn, medium vertical bar extended strand (**c**) predicted conserved domain for the protein encoded by TSEG2. (**d**) Estimated three-dimensional structures of the protein encoded by TSEG2.

**Figure 4:**
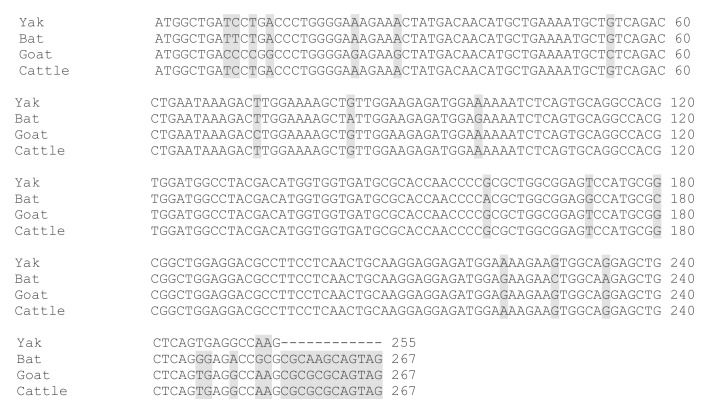
Multiple alignment of full length sequences of yak TSEG2 protein with bat, goat, and cattle. Dissimilar amino-acids are represented by shaded boxes.

**Figure 5:**
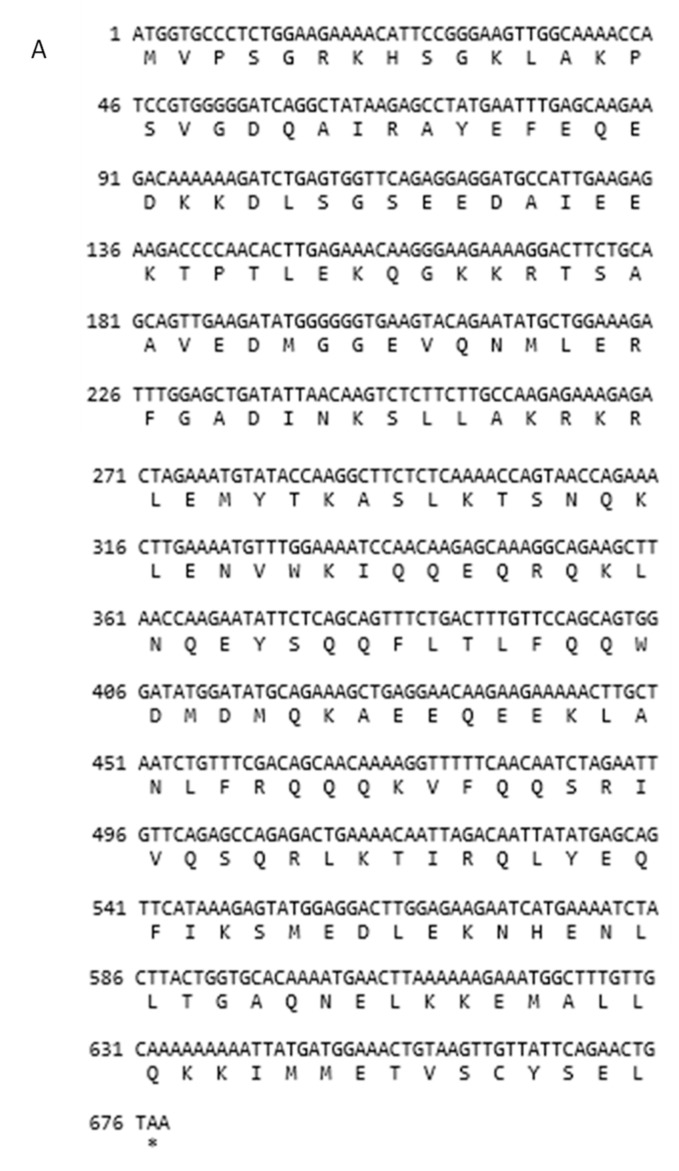

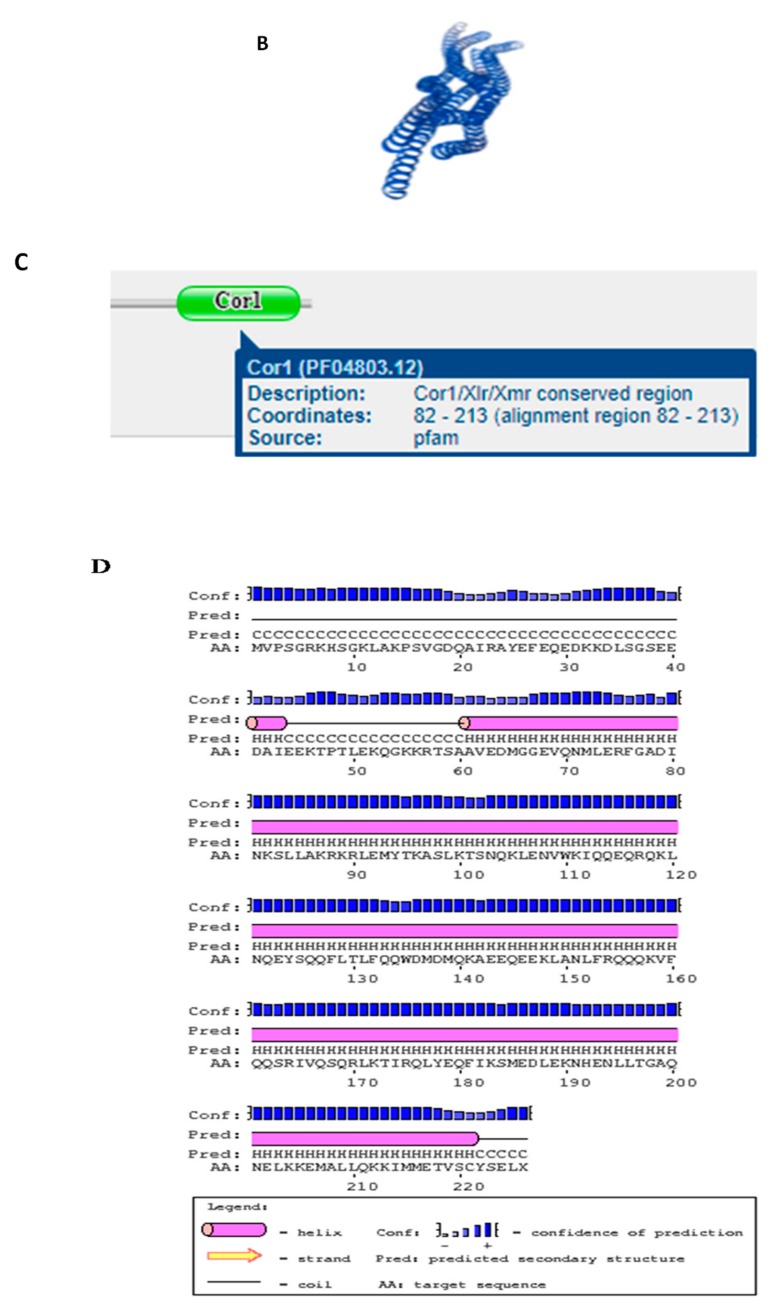
The predicated protein sequence coded by SYCP3 of yak using different bioinformatics tools. The protein encoded by SYCP3 comprised of Cor1/Xlr/Xmr conserved regions. (**A**). The predicated protein sequence from yak SYCP3 gene. (**B**) projected three dimensional structures of SYCP3. (**C**) anticipated conserved domain for the protein encoded by SYCP3. (**D**) estimated secondary structures of SYCP3.

**Figure 6:**
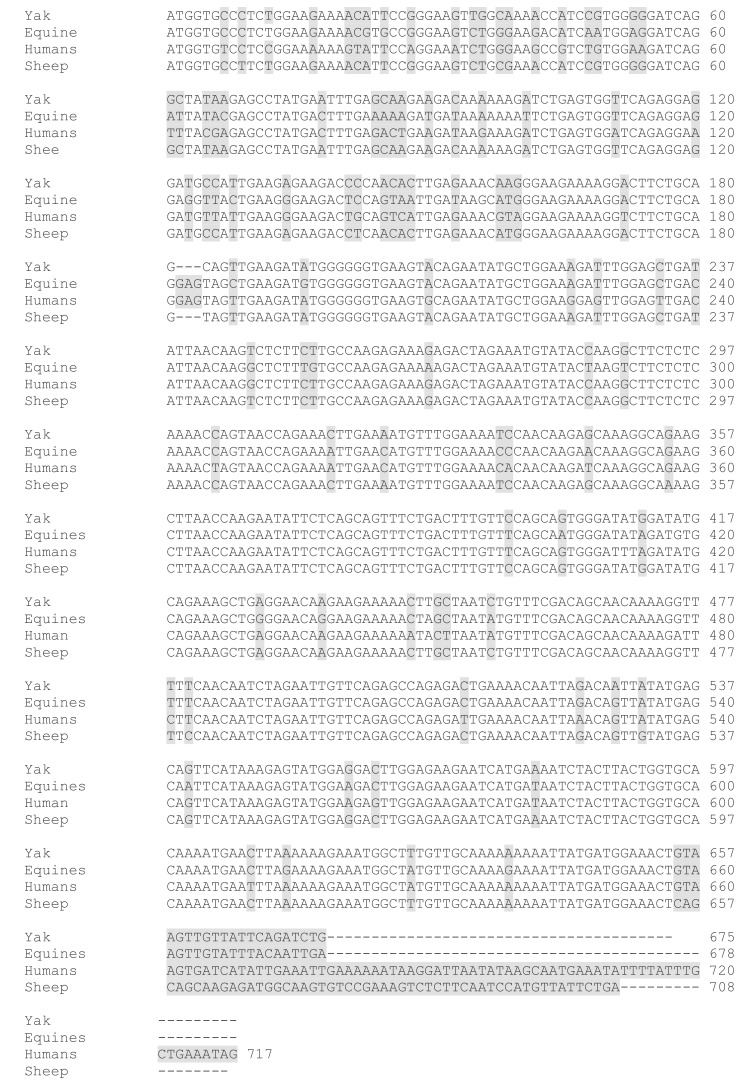
Multiple alignment of full-length sequences of yak SYCP3 protein with equines, humans, and sheep. Dissimilar amino-acids are represented by shaded boxes.

We cloned the coding region of *SYCP3* and *TSEG2* from yak testis. After that, the open reading frame finder program (http://www.ncbi.nlm.nih.gov/gorf/gorf.html) was implemented to predict the amino acid sequence of SYCP3 and TSEG2. While the conserved domain of SYCP3 and TSEG2 protein was anticipated by using Pfam (http://pfam.janelia.org/). The three dimensional and secondary structures for the protein encoded by SYCP3 and TSEG2 were examined by using the PDB viewer, PSI pred (http://bioinf.cs.ucl.ac.uk/psipred) and SWISS-MODEL (http://swissmodel.expasy.org/). The coding region sequences of *TSEG2* gene in yak encoded 88 amino acids (Figure 3a). The protein encoded by *TSEG2* in yak contained a synaptonemal complex central element protein 3 (Figure 3d), and the secondary structures were mainly extended strand and consist of coils and helix (Figure 3b). The three-dimensional structures of the TSEG2 protein were determined to a 1.9 Å resolution by using SWISS-MODEL (Figure 3c). This partial sequence of the TSEG2 to corresponding homologous regions of cattle, goat and bat, shared 100%, 96.44%, and 95.98% sequence identity, respectively (Figure 4). Furthermore, the predicted coding region sequences of *SYCP3* gene encoded a protein comprised of 225 amino acids residues (Figure 5A). The crystal structures of SYCP3 were elucidated by X ray diffraction 2.24 Å and oligo state homo-tetramer (Figure 5B). On the other hand, the protein encoded by SYCP3 in yak comprised of Cor1/Xlr/Xmr conserved regions (Figure 5C). The predicted secondary structure of the protein encoded by *SYCP3* gene was consisting of helix, extended strand, and coil (Figure 5D). While multiple alignment sequence of SYCP3 to corresponding homologous regions of Equine, humans, and Sheep share 88.41%, 85.62%, and 98.36% sequence identity, respectively (Figure 6). Hence, from this observation we reported that the protein encoded by Datong yak SYCP3 and TSEG2 were highly conserved among mammalians. 

### 3.3. Tunel Assay Analysis 

**Figure 7:**
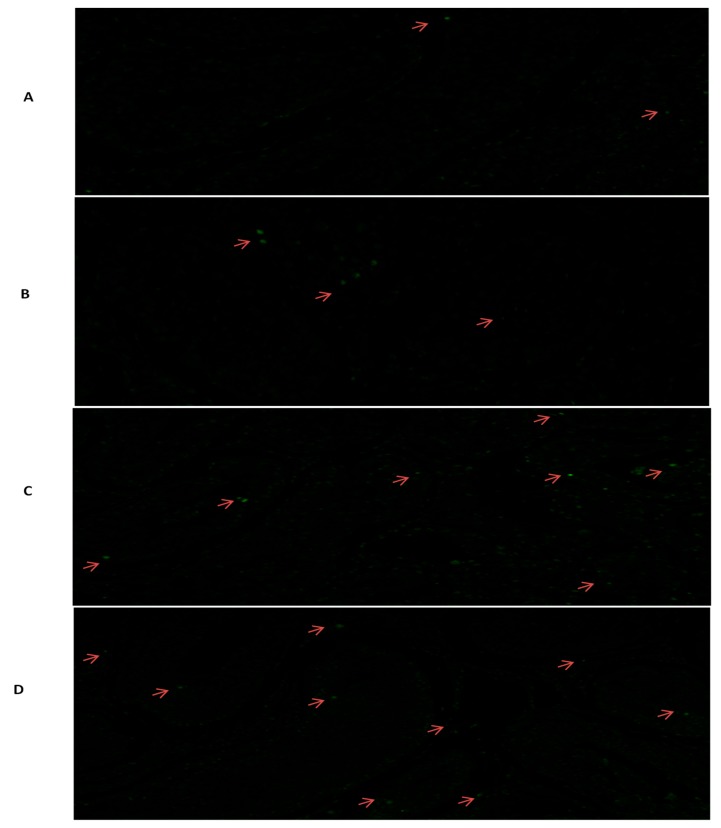

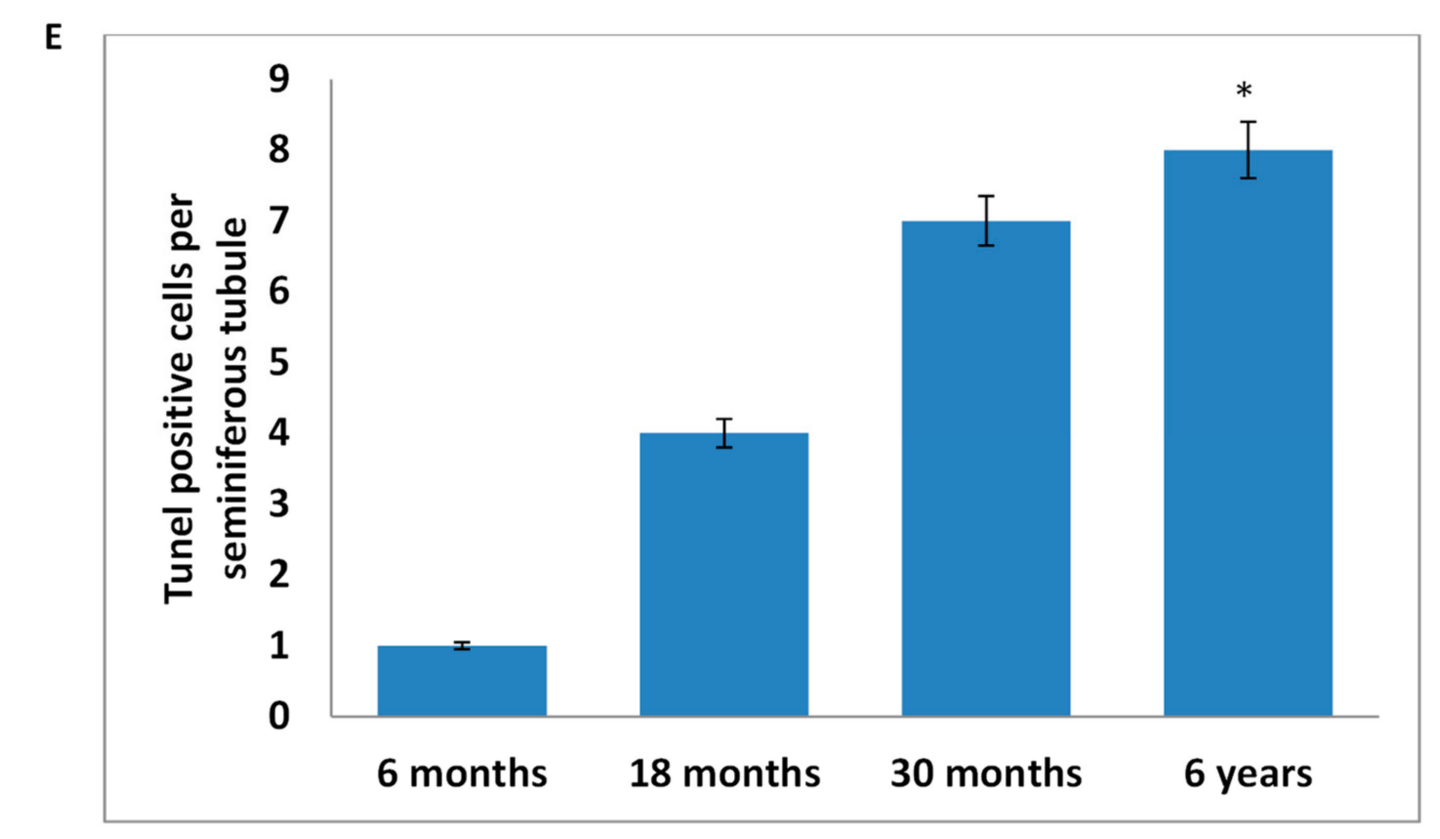
Tunnel assay of testis of different ages of Datong yak. (**A**) 6 months. (**B**) 18 months. (**C**) 30 months. (**D**) 6 years. TUNEL-positive germ cells were rare in 6 months testes. The TUNEL-labeled apoptotic cells were counted, and the apoptosis rates were determined by calculating the average percentage of the total cells per seminiferous tubule. The symbol (*) indicated a significant increase from 6 months (<0.05).

Apoptosis of the spermatogenic cells in different developmental ages are presented in Figure 7. These results specified that apoptosis cells all present in all ages but they are higher in 6 years followed by 30, 18, and 6 months testis, respectively. Besides, in 6 years testis apoptosis were significantly higher than 6 months, while non-significant difference was observed in other ages. Additionally, apoptotic cells were not detected in spermatids located close to the lumen. Whereas, large apoptotic pachytene spermatocytes can be clearly recognized and they are represented by red arrow.

### 3.4. Western Blotting Analysis 

**Figure 8:**
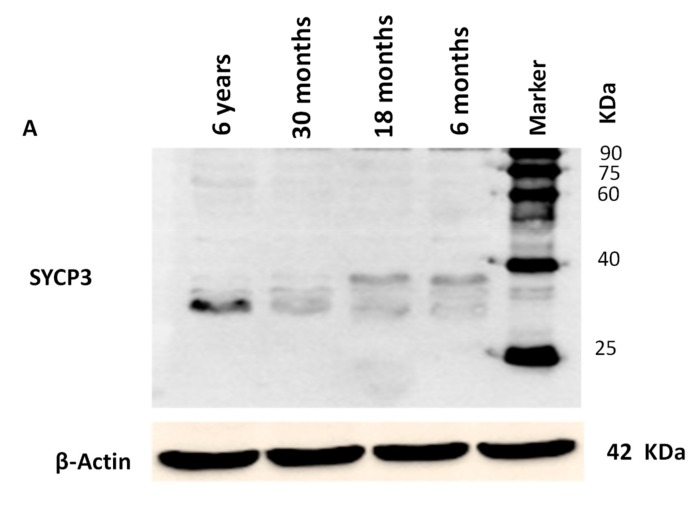

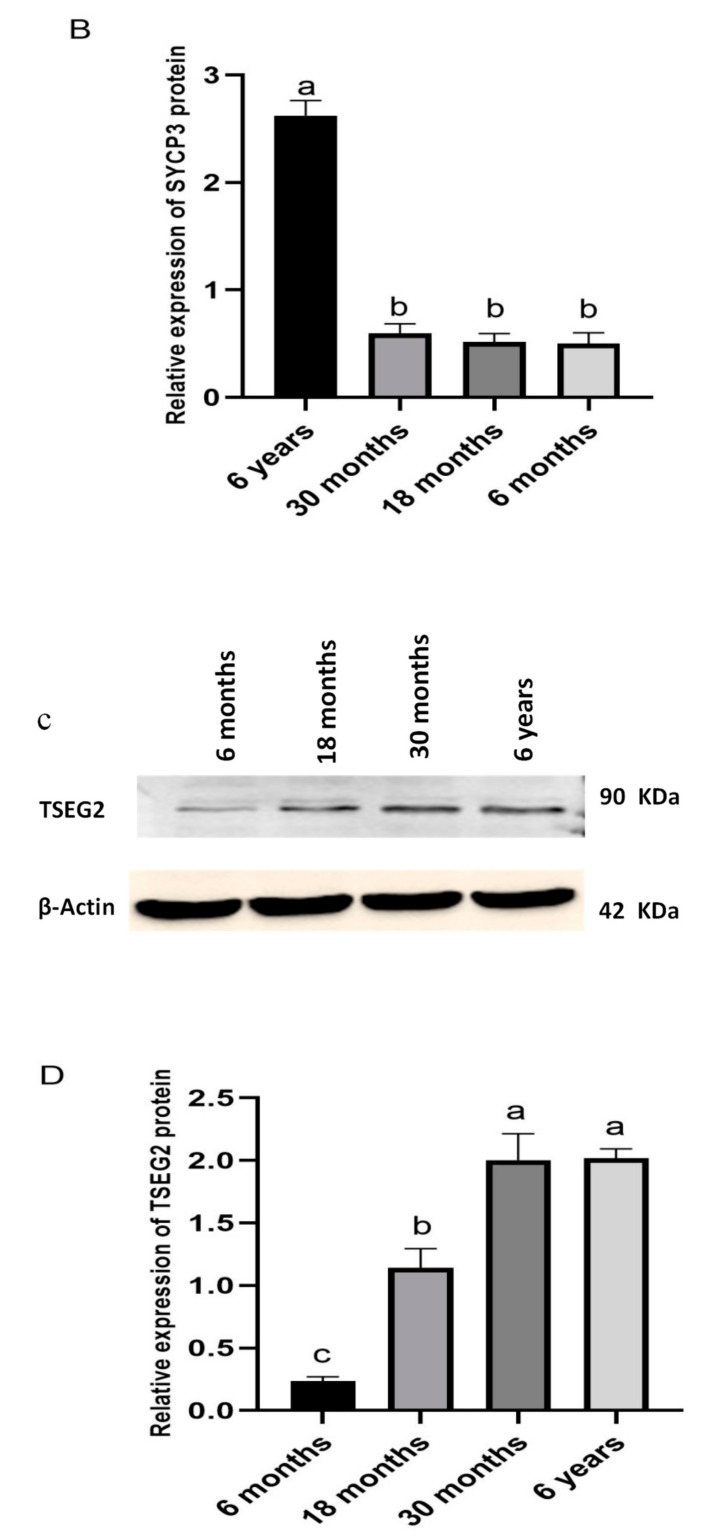
Characterization of SYCP3 and TSEG2 proteins in the testis of Yak. (**A**) Western blot results of SYCP3. (**B**) Relative expression of SYCP3 protein. (**C**) Western blot results of TSEG2. (**D**) Relative expression of TSEG2 protein from testes, while Beta actin was used as control. Protein levels were quantified by densitometric analysis. Different letters indicate significant difference (*p* < 0.05).

Western blot analysis was also performed for the determination of SYCP3 and TSEG2 protein in different ages of Datong yak testis (Figure 8). These findings represented that the SYCP3 and TSEG2 proteins were present in yak testes. As we compared the testes of different ages, we evaluated that there was a significant differential expression of SYCP3 and TSEG2 protein, the SYCP3 protein was prominent in 6 years and was low in expression in other ages (Figure 8A). While, TSEG2 protein was higher in 6 years and 30 months and moderate in 18 months followed by 6 months (Figure 8B). Moreover, a 34-kDa (SYCP3) and 90-KDa (TSEG2) proteins were detected from the testicles of yak.

### 3.5. Immunostaining Analysis

**Figure 9:**
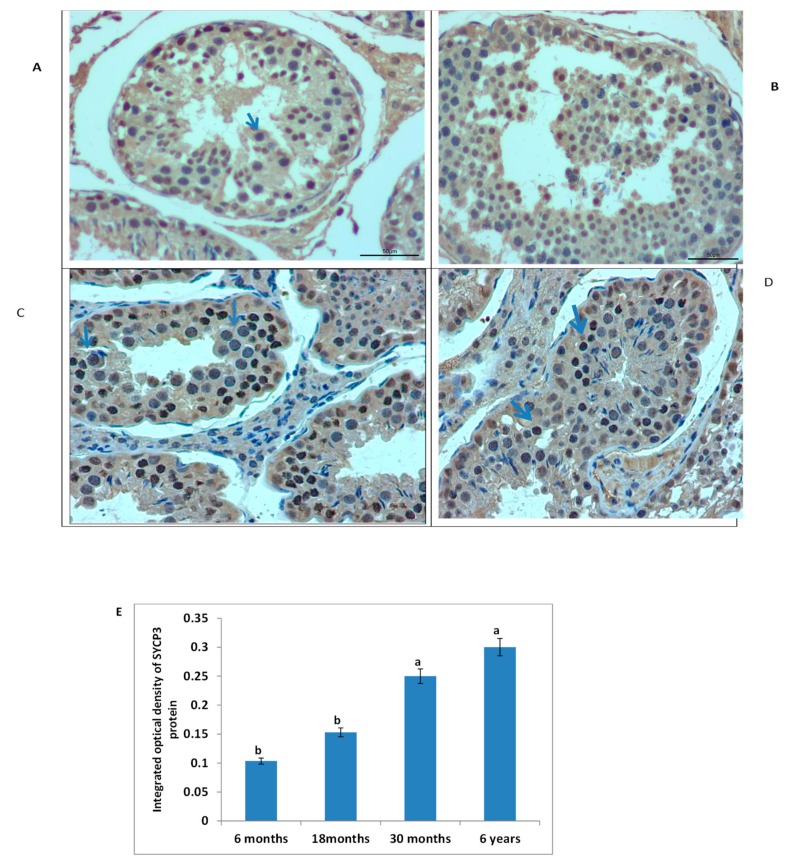
Immunostaining of testis of various ages of Yak. Immunostaining analysis showed that SYCP3 is present in all ages but positive cells (blue, arrow) are more abundant in 6 years and 30 months yak testis as compared to 18 and 6 months respectively. (**A**) 6 months, (**B**) 18 months, (**C**) 30 months, (**D**) 6 years. Scale bar 50 µm. (**E**) Integrated optical density of SYCP3 protein. Different letters indicate significant difference (*p* < 0.05).

Furthermore, we invesigated the morphological differences in different ages of yak testis using immunostaining analysis (Figure 9). From immunostaining analysis, we observed that SYCP3 was present in all tissues but positive cells (blue arrow) were prominant in the tubules of 6 years and 30 months as compared to 6 and 18 months respectively. Adtionally, intergral optical density of SYCP3 also showed that SYCP3 was significantly higher in 6 years and 30 months (P < 0.05). Therefore, these results underlined that the appearance of SCP3-positive protein may be play prominent role in the maturation of spermatocytes. 

## 4. Discussion

It has been speculated that many genes are involved in normal process of spermatogenesis and male fertility but when mutations of these genes occur it causes spermatogenic disorder and infertility. The *SYCP3* and *TSEG2* genes are key structural component of synaptonemal complex and they play crucial role spermatogenic cell development and in fertility [9]. Moreover, fertility is more important in an individual bull than an individual cow, because one bull may be used to breed up to 40 females with natural service, or potentially hundreds of thousands via artificial insemination [25]. In humans, infertility mostly occurs due to high aneuploidy rate in oocytes [26] and failures of the synaptonemal complex [27]. Previous studies elucidated that *SYCP3* and *TSEG2* genes have a crucial role in male fertility and in spermatogenesis, but still no research was available regarding SYCP3 and TSEG2 genes in male yaks, therefore this research was planned in yaks of different ages to confirm the significance of *SYCP3* and *TSEG2* in male yak fertility. So in this research, we cloned the coding regions of bovine *SYCP3* and *TSEG2* genes, and our results indicated that *TSEG2* cDNA sequence contained a 328 bp that encoded a protein of 88 amino acids. Furthermore, the predicted coding region sequences of *SYCP3* gene encoded a protein comprised of 225 amino acids residues. Moreover, multiple alignment sequence showed that there was a high level of sequence identity between *SYCP3* and *TSEG2* genes of yak with other animals (Figure 4 and Figure 6). Similarly one previous research specified that these were highly conserved among mammals with an identity of 90% (96% similarity) at the amino acid level between mouse and human [9]. It represents that *SYCP3* and *TSEG2* genes were highly conserved among the mammals.

Our findings of semi quantitative PCR and qPCR elucidated significantly high mRNA expression levels of *SYCP3* gene at 6 years and 30 months in testes as compared to other tissues. Similarly, Aarabi et al. [28] evaluated the higher occurrence of SYCP3 in testis of human and suggested that the lack of expression of SYCP3 in testis had harmful effect on fertility and spermatogenesis. Moreover, the high mRNA expression level of *SYCP3* is corresponding to the work of Syrjänen et al. [1,13]. Additionally, consistent with these results, Wang et al. [20] showed the significantly higher expression level of bovine *SYCP3* mRNA in testes of cattle. This implies that bovine SYCP3 plays an important role in meiosis of yak spermatogenesis. Moreover, Yuan et al. [18] reported that SYCP3 deficient male mice failed to form synaptonemal complexes, axial/lateral elements and the chromosomes in the mutant spermatocytes did not synapse. The high expression of the *SYCP3* gene in the testis of yak may show that *SYCP3* gene is important for male fertility and in the development of testis.

In a previous study Wang et al. [16] elucidated *TSEG2* gene expression in the testes of mice at different growing stages. For more clearance in this study, we also examined the characterization of TSEG2 from different tissues of yak by semi quantitative PCR. The findings of the semiquantitative PCR indicated that the expression levels of TSEG2 mRNA was higher in the testis of yaks. Our outcomes are analogous with the judgments of Hu et al. [15]; their study represented that expression of *TSEG2* gene was higher in the testis, and finally they concluded that the *TSEG2* gene was involved in the apoptosis of spermatogenic cells and up regulated in established cryptorchidism models. Furthermore, we also compared the expression level of TSEG2 mRNA by performing quantitative PCR in different tissues of Yak. These results characterized the expression level of TSEG2 mRNA in different organs but it was significantly higher in testis as compared to other organs. Our results are consistent with Wang et al. [16]: their conclusions showed that TSEG2 was present in the cytoplasm of spermatogonia and spermatocytes. Finally, we assumed that TSEG2 was particularly present in testis and its appearance during different developing stages of testis relates with sexual maturation and spermatogenesis.

Additionally, the protein encoded by yak SYCP3 contains Cor1/Xlr/Xmr conserved regions (Figure 5C); similarly, another study revealed that SYCP3 protein has a classic conservative motif, the Cor1 motif (Cor1⁄Xlr⁄Xmr conserved region) in bovines [29]. Moreover our findings presented that the sequence similarity among all mammalian was 70% which was predicated using SWISS-MODEL. This is in consistent with the findings of [20] who described the amino acid sequence similarity of SYCP3 Cor1 motif between bovines and other mammals, from 72% to 88%, and demonstrated that it played a significant role in the process of meiosis within the spermatocytes. Consistently, the work of [28] reported that a secondary functional member of the Cor1 ⁄ Xlr ⁄Xmr was abundantly transcribed in the testes in a tissue-specific and developmentally regulated manner. Earlier studies also revealed that bovine *SYCP3* gene played a great contribution in the process of meiosis and spermatogenesis [20]. The predicted secondary structure of the protein encoded by *SYCP3* gene in yak was consisting of the helix, extended strand, and coil (Figure 5D). Comparable to these results, [13] reported the crystal structure of SYCP3 located at both ends of the central helical core and contained tetrameric form with N terminal DNA binding domains. Estimated secondary structures of TSEG2 presented that they form helix like structures (Figure 3b). One previous research also indicated that TSEG2 contains helix-like structural component and initiate along with synapsed chromosomes in pachytene and zygotene spermatocytes, which displayed that TSEG2 in SC assembly might function as higher order oligomer or dimer in cells [14]. An emerging depiction is that synaptonemal complex proteins are classified as either central element or transverse filaments appears slightly uninformed, as these protein equally contributed in stabilization, organization of transverse filament, as well as synaptonemal complex assembly, central element formation, and generating a conserved structure with similar dimensions in different organisms [30]. Moreover, the coding region sequences of *TSEG2* gene in yak encoded 88 amino acids (Figure 3a). Similarly Schramm et al. [9] revealed that SYCE3 (TSEG2) consisting of 88 amino acids and that it can be found in all vertebrate. Besides, the predicted three dimension structures of TSEG2 protein showed that these are consists of C-helix and N-helix (Figure 3c). Schmekel et al. [31] suggested that bimodal distribution patterns for the N-terminal region of SYCE3 associated with the transverse filaments within the central element of the synaptonemal complex. In addition to this SYCE3 proteins are required for stabilization of the N-terminal region of SYCP1 within the central element of the synaptonemal complex and for synaptonemal complex extension; SYCE3 knockout mice exhibit both male and female infertility [7,8]. This indicates that both the three-dimensional structure and biological function are conserved.

Furthermore, the western blot analysis indicated that molecular mass of SYCP3 proteins from testis tissue was 34-kDa in the yak. Comparable to these results, [32] reported that the molecular mass SYCP3 protein was 30 kDa. Meanwhile, Izadyar et al. [33] determined 33 kDa band of SYCP3 in cultured spermatogonia at 60 and 100 days. On the other hand our findings reported that the molecular weight of TSEG2 protein in Yak was 90 kDa. However in another study they reported molecular mass of TSEG2 protein was 20.5 kDa in Transfected COS-7 cells. In addition Schramm et al. [9] detected 12 kDa molecular weight of TSEG2 proteins in mouse testes in different ages.

Immmunohistochemistry (IHC) examination directed that SYCP3 was present in all samples but positive cells (blue arrow) are greater in 6-year-old and 30-month-old yak testes as comparased to 18- and 6-month-old (Figure 9). Although comparable to these observations, Ozaki et al. [34] exposed that SYCP3 was especially present in spermatocytes at each meiotic stage in zebra fish in typical nuclear patterns. In agreement with current results, [35] suggested that SYCP3 like Y-linked was involved in the development of the acrosome and in the process of spermatogenesis. Moreover, mutations and polymorphism of the *SYCP3* gene in women was possibly associated with recurrent pregnancy loss [6,36]. Shi et al. [21] recommended that SYCP3-like X-linked 2 was associated with DNA recombination, synaptonemal complex formation, and sex body maintenance during meiosis, while Aarabi et al. [28] showed that the degree of azoospermatism is associated with low expression level of *SYPC3* gene. Also, the absence of SYCP3 results in Azoospermia, suspended spermatogenesis, and male infertility [18,19]. Moreover apoptosis of the spermatogenic cells in different developmental ages are presented in Figure 7. These results specified that the 6 years and 30 months testis contained a significantly larger number of apoptotic cells than those in 18 and 6 months. While Hu et al. [15] studied the spermatogenic cells apoptosis in cryptorchid testis and assumed that heat stress might enhanced the expression of TSEG2 to excite the spermatogenic cells apoptosis. These findings showed that TSEG2 may play a part in the apoptosis of spermatogenic cells and in the pathogenesis of cryptorchidism. 

## 5. Conclusions

In summary, our results showed that the *SYCP3* and *TSEG2* genes were exclusively higher in male germ cells as compared to other organs and expression of both genes increased when animals were going towards sexual maturity. Thus, current data indicate that *SYCP3* and *TSEG2* might play an important role in the process of spermatogenesis, maturation of seminiferous tubules, and in promoting the maturation of spermatocytes. Moreover, the higher expression of TSEG2 in testis through tunnel assay analysis revealed that *TSEG2* may participate in the apoptosis of spermatogenic cells. These findings provide a novel genetic explanation for further understanding of the role of *SYCP3* and *TSEG2* genes in spermatogenesis and male infertility in other animals during non-breeding season. 

## Figures and Tables

**Table 1:** Primers for qPCR and cloning were designed based on bovine genomic sequences.

Accession no	Gene	Primers Sequence (5’- >3’)	Product Length (bp)	Annealing Temperature (°C)
NM_001206333.1	TSEG2 (For cloning)	F: TGGCAGGTAGCTGAACAGGA R: GTTGGGGTCGCTGTGGTTC	328	60.84 61.26
XM_005903404.1	TSEG2 (For gene expression)	F:CTGACCCTGGGGAAAGAAACT R: ACCATGTCGTAGGCCATCCA	130	59.57 60.98
XM_024992196.1	SYCP3 (For cloning)	F: TACGCCTGTCCGGAGACATT R: GACTTTCGGACACTTGCCATC	942	60.97 59.54
XM_019961171.1	SYCP3 (For gene expression)	F:TCCGGGAAGTTGGCAAAACC R: GGTCTTCTCTTCAATGGCATCC	117	61.11 59.05
NM_001034034.2 F: AATGAAAGGGCCATCACCATC	GAPDH	F:AATGAAAGGGCCATCACCATC R:CACCACCCTGTTGCTGTAGCCA	204	55.85 60.00
